# Supplementary material for: Allosteric activation of vinculin by talin
Source: Nat Commun. 2023 Jul 18;14:4311. doi: 10.1038/s41467-023-39646-4 (PMC10354202; doi:10.1038/s41467-023-39646-4)
Supplement: Supplementary file 1 — Supplementary Information [file 41467_2023_39646_MOESM1_ESM.pdf]

Supplementary Information

Allosteric Activation of Vinculin by Talin

Florian Franz[1,2†], Rafael Tapia-Rojo[3,4†*], Sabina Winograd-Katz[5], Rajaa Boujemaa-Paterski[6], Wenhong Li[5], Tamar Unger[7], Shira Albeck[7], Camilo Aponte-Santamaria[1,2], Sergi Garcia-Manyes[3,4], Ohad Medalia[6*], Benjamin Geiger[5*], Frauke Gräter[1,2,8*]

1 Heidelberg Institute for Theoretical Studies (HITS), Schloß-Wolfsbrunnenweg 35, 69118 Heidelberg, Germany;
2 Interdisciplinary Center for Scientific Computing (IWR), Heidelberg University, Mathematikon, INF 205, 69120 Heidelberg, Germany;
3 Department of Physics, Randall Centre for Cell and Molecular Biophysics, Centre for the Physical Science of Life and London Centre for Nanotechnology, King's College London, Strand, WC2R 2LS London, United Kingdom
4 Single Molecule Mechanobiology Laboratory, The Francis Crick Institute, 1 Midland Road, London NW1 1AT, London, UK.
5 Department of Immunology and Regenerative Biology, Weizmann Institute of Science, Rehovot, Israel;
6 Department of Biochemistry, University of Zurich, 8057 Zurich, Switzerland;
7 The Dana and Yossie Hollander Center for Structural Proteomics, Weizmann Institute of Science, Rehovot, Israel;
8 IMSEAM, Heidelberg University, INF 225, 69120 Heidelberg, Germany;

†contributed equally
*frauke.graeter@h-its.org, rafael.rojo@kcl.ac.uk, benny.geiger@weizmann.ac.il, omedalia@bioc.uzh.ch

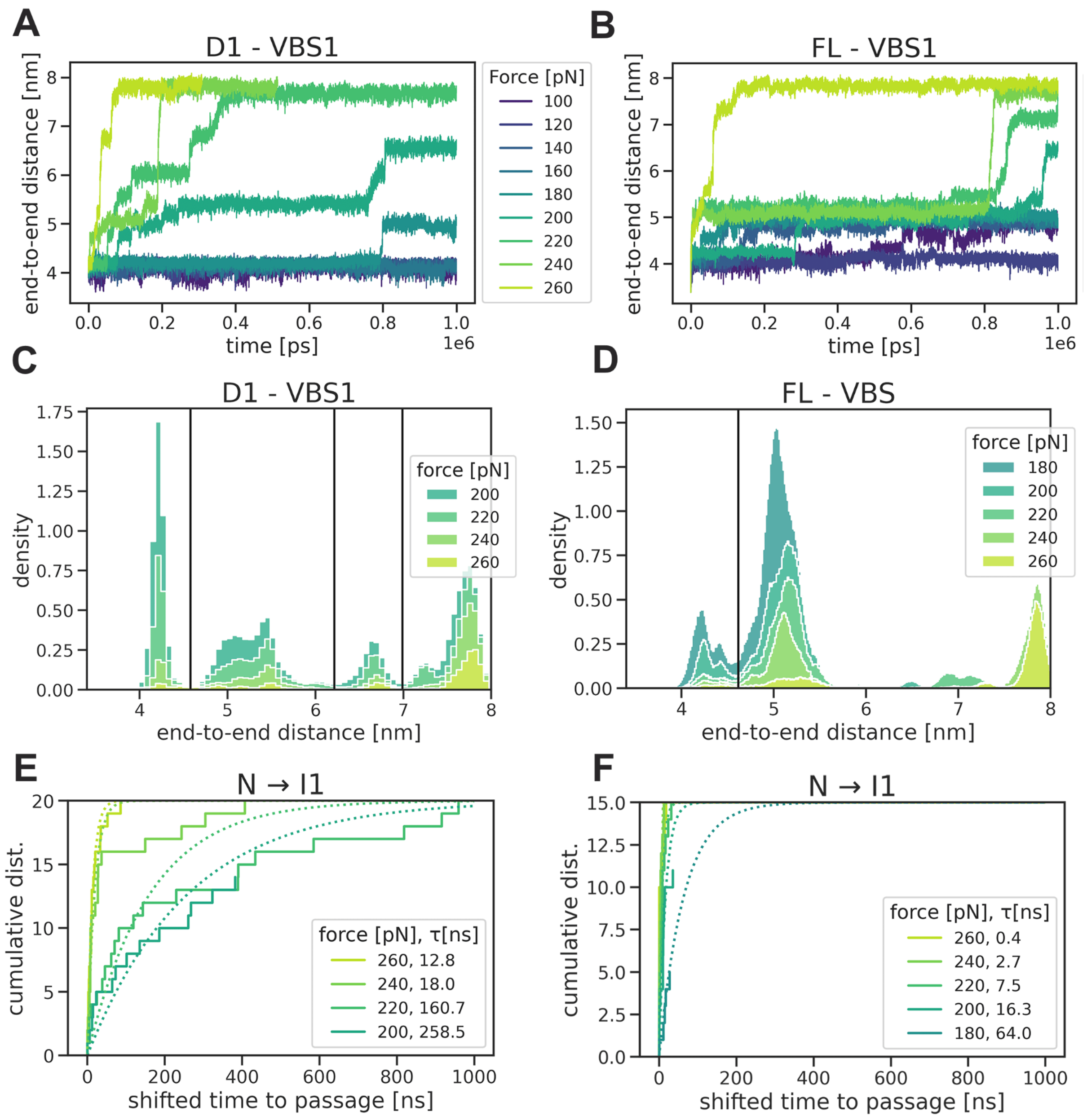

Supplementary Figure 1: Kinetic analysis of the force-probe MD simulations of VBS1 bound to D1. (A,B) End-to-end distance traces for the pulled VBS1 peptides in complex with the Vinculin D1 domain (**A**) and the full protein (**B**) for forces between 100 and 260 pN. **(C,D)** Stacked distributions of end-to-end lengths observed during 1 µs-long MD simulations at four different constant forces. The locations of the main barriers along the reaction coordinate, identified by a low density of end-to-end distances at that distance, are indicated by the black vertical lines. The initial unfolding step from U (unfolded) to I1, the first intermediate, initiated the full unfolding that results in dissociation. The U->I1 transition was used as a proxy of vinculin-VBS dissociation. **(E,F)** For each force, the dwell time of the unfolded state N to I1 was recorded and here the cumulative distribution of these dwell times is shown. Data were fit with a single exponential function to deduce the VBS unfolding time (denoted mean first passage time, MFPT, in the main text) for each given force (inset).

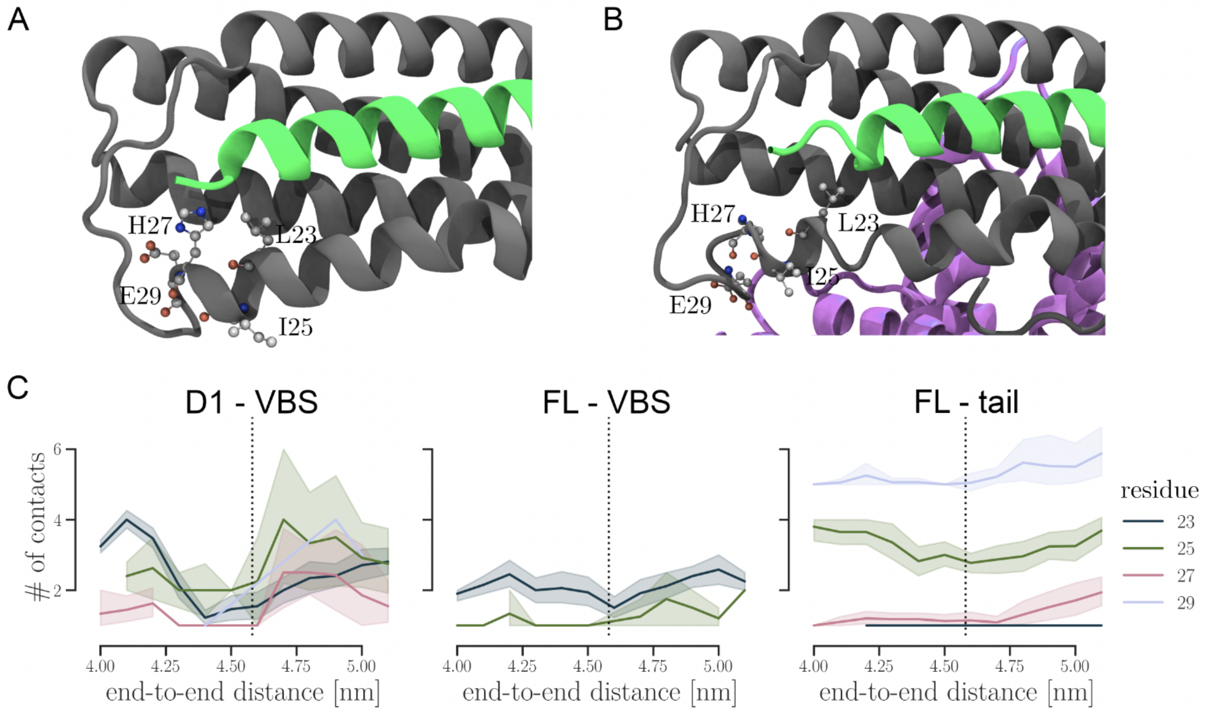

Supplementary Figure 2: Vinculin tail and talin VBS compete for the interaction with D1. **(A)** Cartoon representation of the vinculin D1 domain (grey)-VBS1 (green) interaction. Residue L23 and H27 reinforce the complex. **(B)** With full-length vinculin, L25 and E27 orient towards the vinculin tail (purple). **(C)** The evolution of selected inter-residue contacts with respect to the VBS end-to-end distance. Left) D1 residues in contact with VBS for the D1-VBS1 complex. Center) Residues in contact with VBS when simulating the full-length vinculin complex. Right) The number of contacts between D1 and the tail domain. The solid lines show the average obtained from up to 60 simulations. Shaded area represents the standard deviation.

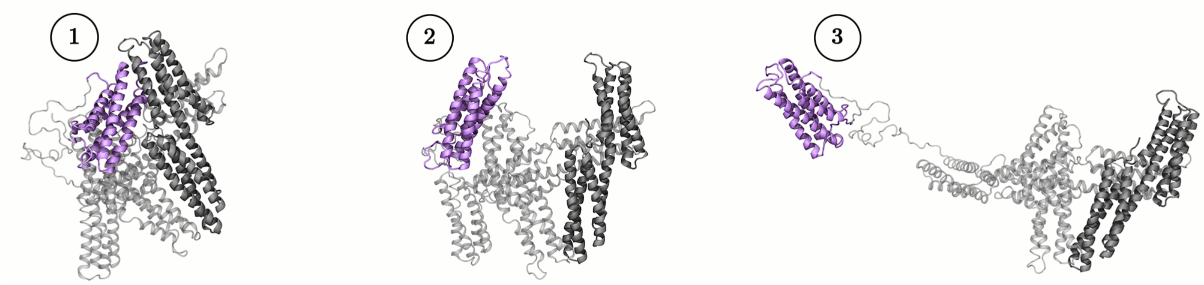

Supplementary Figure 3: Snapshots of head-tail dissociation in apo-vinculin as sampled by FPMD simulations. Coloring as in Fig. 2A of main text.

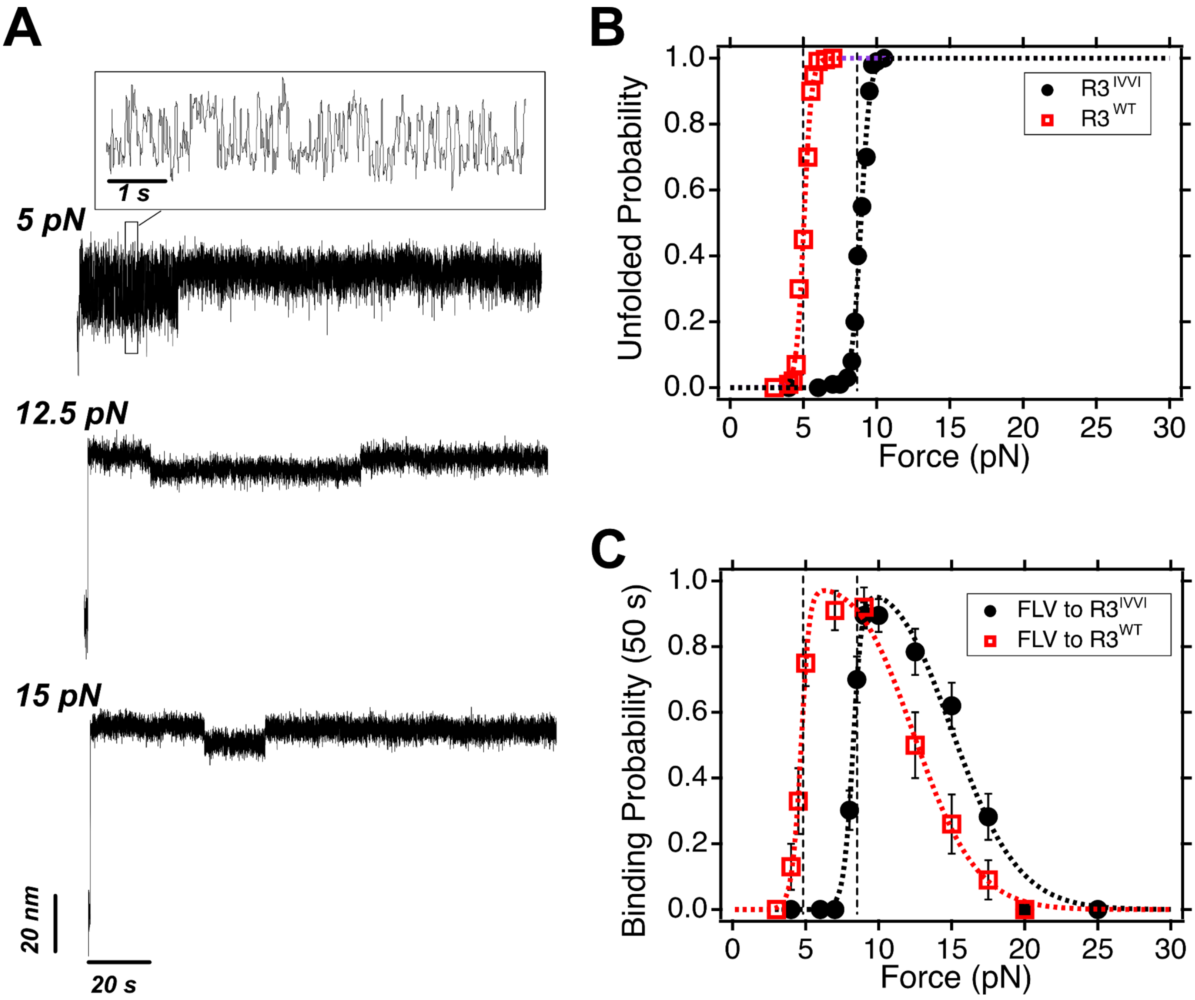

Supplementary Figure 4: Full-length vinculin binding to the R3^{WT} domain. (A) Magnetic tweezers recordings of R3^{WT} in presence of 20 nM full-length vinculin measured at different forces. As previously found for the Vd1 domain, vinculin binds similarly to R3^{WT} and to R3^{IVVI}, only showing a lower threshold force for binding due to the lower mechanical stability of the WT domain. **(B)** Probability of populating the unfolded state for R3^{IVVI} (black circles) and R3^{WT} (red squares). The IVVI mutation increases the mechanical stability of the R3 talin domain, shifting the coexistence force (dotted vertical lines) from ~5 pN (R3^{WT}) to ~9 pN (R3^{IVVI}). Data from N>10 molecules. **(C)** Binding probability of full-length vinculin to R3^{IVVI} (black circles) and R3^{WT} (red squares) measured over a 50 s time-window. The threshold force for binding is lowered from ~9 pN (R3^{IVVI}) to ~5 pN (R3^{WT}) correlates with the unfolded probability and, hence, the exposure of the cryptic vinculin binding sites. Besides this difference, the probability of vinculin binding to either R3^{WT} or R3^{IVVI} shows a similar force dependence, suggesting a comparable binding mechanism. Data from N=207 binding pulses measured on 5 R3^{WT} molecules. Error bars are SEM.

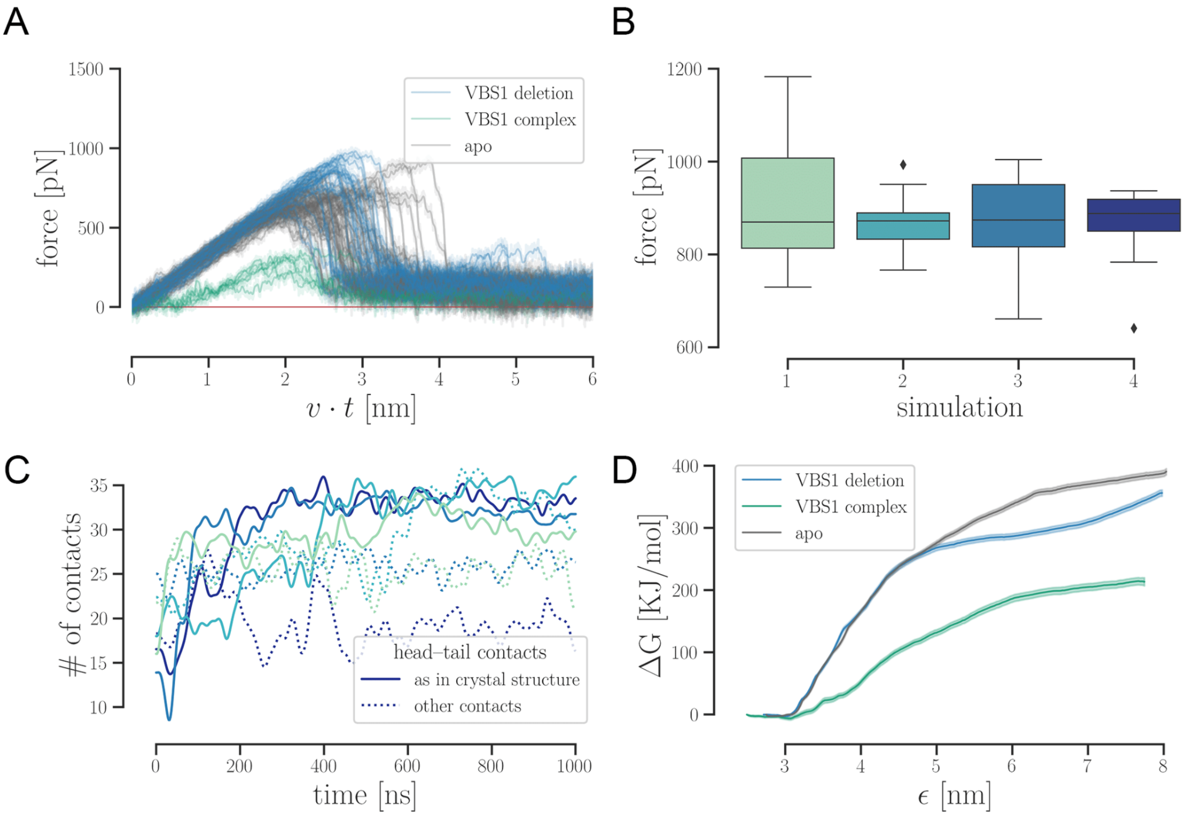

Supplementary Figure 5: Loosening of the head-tail interface is caused by VBS binding and not the use of different starting crystal structures, as shown by additional equilibrium, FPMD, simulations not started from the apo crystal structure but instead from the VBS-vinculin crystal structure, from which VBS was removed and vinculin equilibrated. **(A)** Force extensions curves for force-probe simulations of the VBS1 complex (green), the apo-state protein (gray) and the 'recovered' apo state after the VBS1 peptide was deleted and the resulting structure was equilibrated. **(B)** Following VBS1 deletion, four independent equilibration runs were carried out. The box plot shows the highest forces observed in 10 pulling simulations starting from each of the relaxed structures. **(C)** The solid lines show the number of contacts that are recovered during the four equilibration simulations. As a reference, we used 53 head-tail contacts identified in the crystal structure with a 3.5 cut-off. Dotted lines show the number of non-apo contacts. **(D)** Mean (solid line) and standard deviation (shaded area) of the free-energy profiles calculated by umbrella sampling for the opening of the described structures.

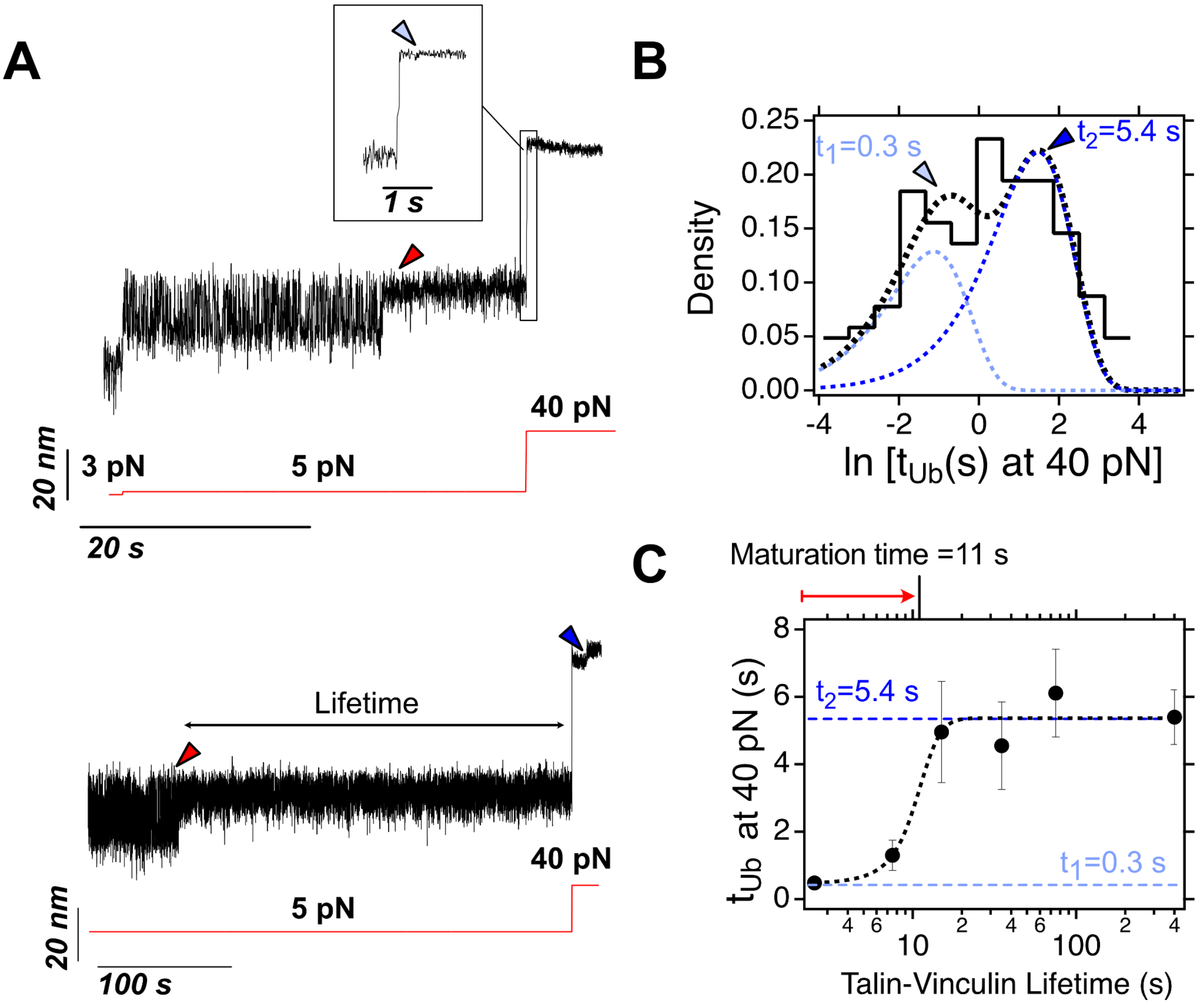

Supplementary Figure 6: Maturation dynamics of the R3^{WT}-full length vinculin complex
(A) Magnetic tweezers recording of full-length vinculin binding R3^{WT} at 5 pN and unbinding upon a 40 pN pulse. If the unbinding pulse is conducted shortly after vinculin binds (upper trace), the complex quickly dissociates (<1 s) as vinculin is expelled by the high force, detectable by a ~3 nm step. However, if the unbinding pulse is applied long after the complex forms (lower panel), the unbinding kinetics are much slower, suggesting that the complex undergoes a maturation process to render a stronger interaction. **(B)** Distribution of unbinding times (t_{Ub}) measured at 40 pN, calculated as a square-root histogram (logarithmic binning). The distribution is bimodal, indicating that there are two unbinding timescales, a fast one of t_1~0.3 s and a second slower one of t_2~5.4 s. The distributions were calculated irrespective of the lifetime of the talin-vinculin complex, so the relative populations of both sub-distributions are arbitrary. Data from *N*=164 unbinding events measured on 5 talin molecules. **(C)** Vinculin unbinding time measured at 40 pN plotted as a function of the talin-vinculin lifetime. The unbinding times show two regimes, a first weak interaction captured at short lifetimes (t_1~0.3 s), and a stronger one achieved over longer lifetimes (t_2~5.4 s), suggesting that the talin-vinculin interaction matures from an initial weakly bound state to a tighter complex. A sigmoidal fit to this dependence permits to estimate the maturation time as ~11 s. The maturation force is 5 pN (coexistence force of R3^{WT}). Data from N=164 unbinding events measured on N>3 talin molecules. Error bars are SEM.

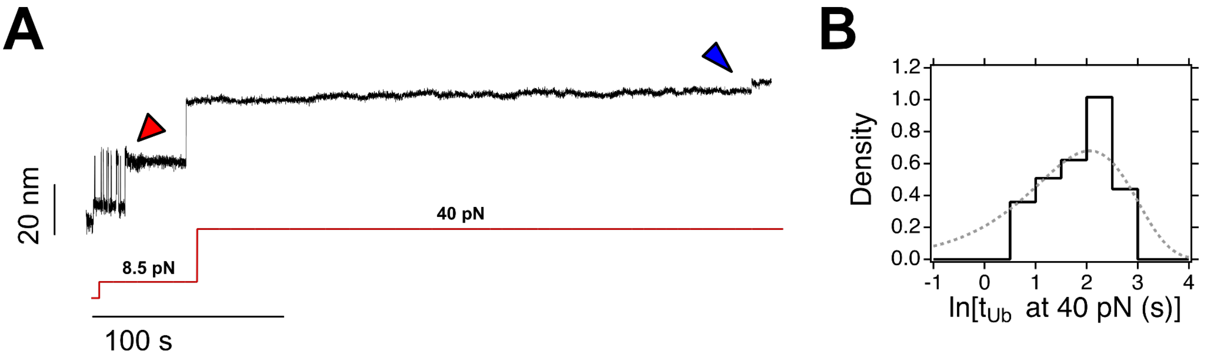

Supplementary Figure 7: Unbinding kinetics of full-length vinculin head. (A) Typical magnetic tweezers recording of full-length vinculin head binding (read arrow) and unbinding (blue arrow) from talin $R3^{IVVI}$. At 20 nM vinculin head, binding at 8.5 pN occurs after a few seconds; however, to dissociate the complex at 40 pN requires a few hundreds of seconds, similar to the Vd1 domain, suggesting that of vinculin head, only the D1 domain participates in the talin-vinculin interaction. **(B)** Square-root histogram of unbinding times (t_{Ub}) at 40 pN for vinculin head. The single-peaked square-root histogram indicates a single-bound mode, similar to Vd1 and unlike full-length vinculin. Data from *N*=32 unbinding events.

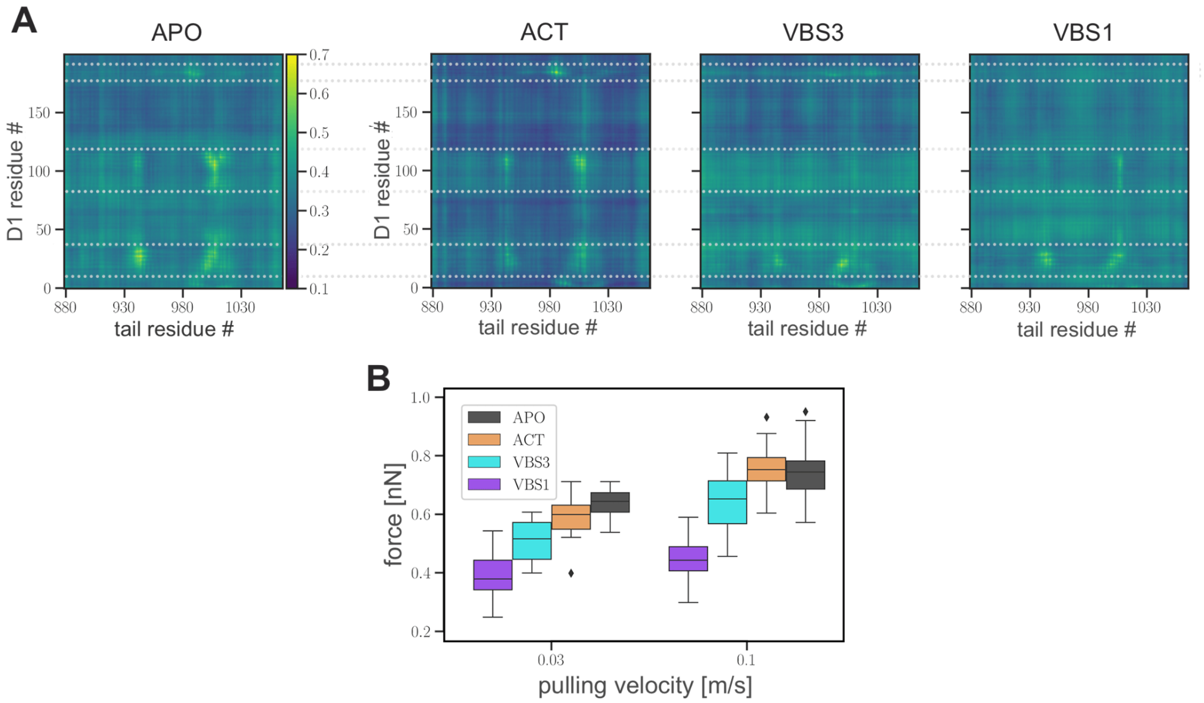

Supplementary Figure 8: Different VBS allosterically affect vinculin head-talin interactions differently. (A) The interactions between vinculin D1 and tail portrayed by generalized correlations maps. The data shown is extracted from force-probe MD trajectories with a pulling speed of 0.1m/s. The correlations were computed for each simulation using a 5 ns-long fraction of each trajectory that precedes the moment of 300 pN force across the protein. The average of 20 runs is presented for each complex type. **(B)** Different VBSs dissimilarly impact the rupture force. The distributions of the highest observed force in each trajectory for a set of simulations ranging over two pulling speeds (0.03 m/s and 0.1 m/s) are represented by a box plot. For each velocity and complex type, the plot represents data from at least 10 replicas.

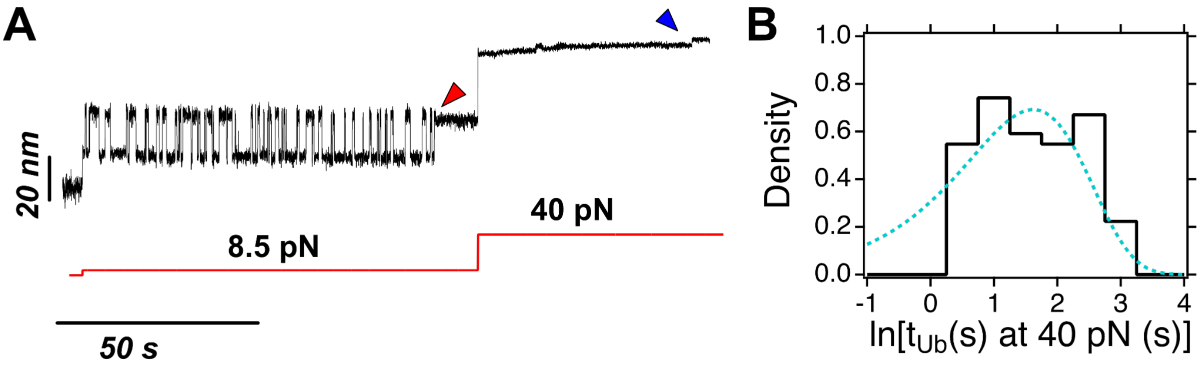

Supplementary Figure 9: Unbinding kinetics of the T12 vinculin mutant. (A) Typical magnetic tweezers recording of T12 vinculin mutant binding (red arrow) and unbinding (blue arrow) from talin R3^{IVVI}. **(B)** Square-root histogram of unbinding times (t_{Ub}) at 40 pN for the T12 vinculin mutant. The single-peaked square-root histogram indicates a single bound mode, similar to the 4M and 5M, also showing a similar unbinding time. Data from *N*=40 unbinding events.

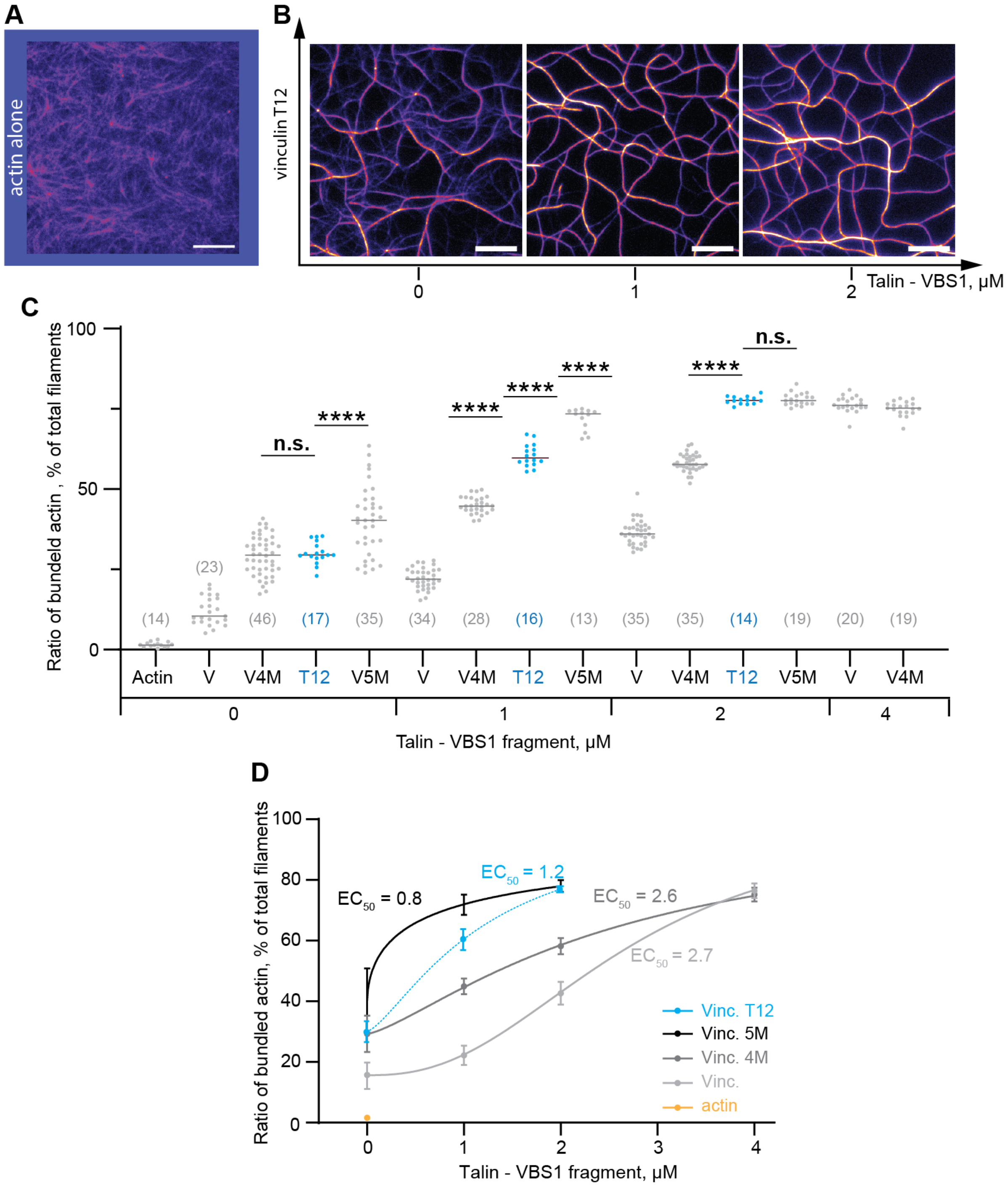

Supplementary Figure 10: Bundling activity of the vinculin T12 mutant. Representative images after 1 hour polymerisation of 0.6 μM Alexa-647 labelled actin alone **(A)** or in the presence 0.5 μM vinculin T12, or in the presence of 0.35 μM vinculin T12 and 1 or 2 μM talin VBS1 **(B)**. Scale bars, 10 μm. **C.** Relative amount of actin bundles produced by Vinculin T12, in comparison to that produced by the WT, V4M, and V5M (showed in **Figure 5**), as a ratio between bundled actin and the total filament population. Statistical comparisons using the Holm-Šídák test and a one-way analysis of variance (ANOVA) showed significant variations between vinculin T12 and V4M and V5M for its ability to generate actin bundles. *NS P>0.05; ****P<10*$^{-4}$. Horizontal bar indicates the average value. (**D**) Counts in C were plotted as a function of talin-VBS1 concentrations and fitted with a four-parameter dose-response equation. Error bars are SD. (see **Methods**).

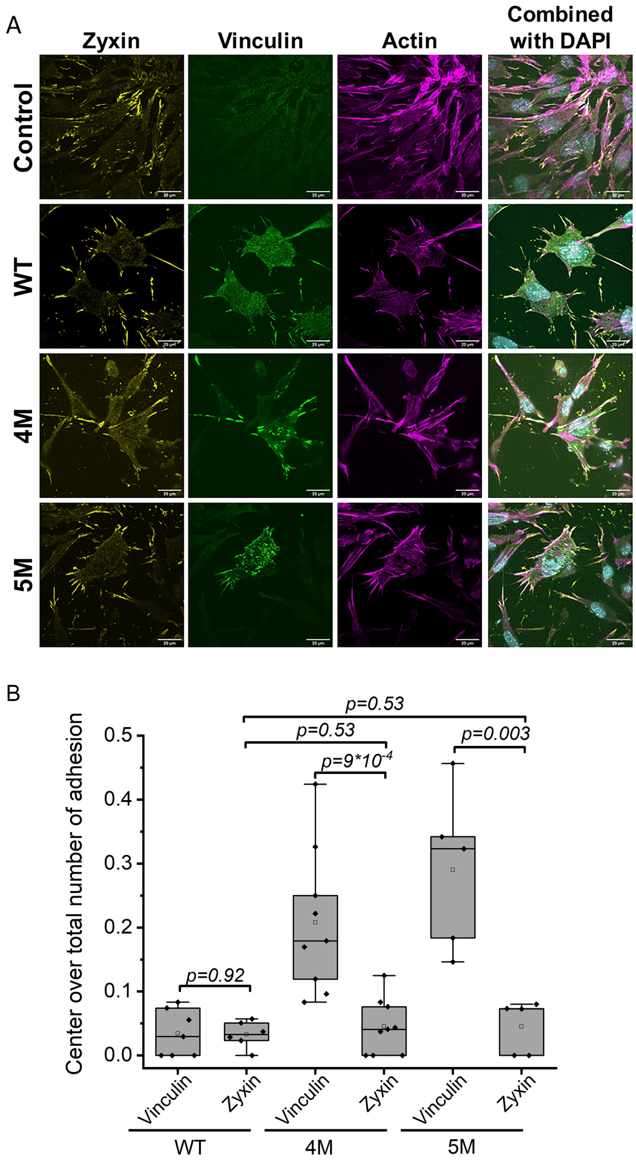

Supplementary Figure 11: 4M and 5M vinculin but not zyxin show a strong propensity to localize in central ("low shear") adhesions. (A) Images of MEF-vinculin-Null cells non-transfected or transfected with His-tag-wild-type vinculin, His-tag-4M, or His-tag-5M vinculin mutants. Cells were immune-stained with anti-Zyxin, anti-Vinculin antibodies, phalloidin and DAPI. (**B)** Quantifications of center vinculin-positive focal adhesions over the total vinculin-positive number adhesions and center zyxin-positive focal adhesion over the total zyxin-positive number adhesions in MEF-vinculin-Null cells transfected with wild-type Vinculin, 4M or 5M Vinculin mutant. The box includes the upper and lower quartile. The lower and upper whisker represents the lower quartile -1.5 *interquartile range and upper quartile +1.5 *interquartile range, respectively. The line in the box represents the median, and the dots represent the mean. Significance levels from two-sided T-tests.

**R3 IVVI - FLV**		**R3 IVVI - D1**	
**Binding**	k_0^F=(1.2 ±0.4)x10^5 s^{-1}	**Binding**	k_0^F=(1.2 ±0.9)x10^5 s^{-1}
	$x^†_F$=5.6± 1.0 nm		$x^†_1$=6.9±2.3 nm
	k_0^U=(5.4 ±0.7)x10^{-5} s^{-1}		k_0^U=(8.7 ±0.9)x10^{-6} s^{-1}
	$x^†_U$=4.7± 1.5 nm		$x^†_U$=4.3±1.6 nm
	k_0=0.11 ±0.09 s^{-1}		k_0=0.22 ±0.011 s^{-1}
	$x^†$=0.09± 0.03 nm		$x^†$=0.31± 0.19 nm
**Unbinding**	k_0=(6.6±0.4)x10^{-3} s^{-1}	**Unbinding**	k_0=(6.8±2.3)x10^{-3} s^{-1}
	$x^†$=0.72±0.6 nm		$x^†$=0.81±0.02 nm

Supplementary Table 1: Parameters for the Bell-Evans fits to the binding and unbinding rates (Fig. 1).

cluster	ID1	ID2	correlation loss
0	185	987	-0.279
1	113	1013	-0.386
2	33	945	-0.289
3	20	1013	-0.262
4	113	1004	-0.296
5	93	1011	-0.247

Supplementary Table 2: Residue pairs with highest correlation loss. The clusters identified in Fig. 4A were scanned for the strongest contributors which are summarized in this table and colored according to Fig. 4B.

Vinc. variant<br><br>Best-fit values	Vinc. WT	Vinc. 4M	Vinc. 5M	Vinc. T12
**a**	15.7	29.3	40.3	30.1
**Hillslope**	2.4	1.3	0.6	1.4
**b**	99.9	99.9	99.8	99.9
**EC_{50}**	2.7	2.6	0.8	1.2

Supplementary Table 3: Best-fit parameters for the dose-response model using the Hill-Langmuir equation (Fig. 5 and S10).

	Number of cells	Number of focal adhesions
**WT**	64	4168
**4M**	62	5580
**5M**	75	6677

Supplementary Table 4: Number of cells and focal adhesions used the quantification
