# Supplementary material for: Allosteric activation of vinculin by talin
Source: Nat Commun. 2023 Jul 18;14:4311. doi: 10.1038/s41467-023-39646-4 (PMC10354202; doi:10.1038/s41467-023-39646-4)
Supplement: Supplementary file 3 — Description Of Additional Supplementary File [file 41467_2023_39646_MOESM3_ESM.pdf]

Description of additional supplementary files

- Supplementary Movie 1: Assembly of actin filaments. Actin filament polymerization in the 291 presences of 0.6 µM actin Alexa-647 labeled, followed by TIRFM. Note that the filaments drift 292 and collide without fusing into bundles. Related Figure 5.
- Supplementary Movie 2: Vinculin 5M mediates stable bundling of actin filaments in the 295 absence of VBS1. Actin polymerization and bundling in the presence of 0.6 µM actin Alexa296 647 labeled, 500 nM of the indicated vinculin variant, followed by TIRFM. Related Figure 5
- Supplementary Movie 3: Vinculin 5M induces increased bundling of actin filaments 299 when supplemented with 1 µM VBS1. Actin polymerization and bundling in the presence of 300 0.6 µM actin Alexa-647 labeled, 350 nM of the indicated vinculin variant, and 1 µM talin-VBS1, 301 followed by TIRFM. Related Figure 5
- Supplementary Movie 4: Vinculin 5M mediates higher degree of actin bundling in the 304 presence of 2 µM VBS1. Actin polymerization and bundling in the presence of 0.6 µM actin 305 Alexa-647 labeled, 350 nM of each vinculin variant, and 2 µM talin-VBS1, followed by TIRFM. 306 Related Figure 5
- Supplementary Movie 5: Vinculin 5M activation by VBS1 is more efficient than vinculin 309 4M and the WT. Actin polymerization and bundling in the presence of 0.6 µM actin Alexa-647 310 labeled, 350 nM of vinculin WT or V4M, and 4 µM talin-VBS1, while 350 nM of vinculin V5M 311 with 2 µM talin-VBS1 yields thicker bundles, followed by TIRFM. Related Figure 5
